# Serum‐derived albumin as a cryoprotective agent for the long‐term storage of red blood cells: A preliminary examination

**DOI:** 10.1111/trf.70192

**Published:** 2026-03-30

**Authors:** Thomas Bailey‐Schmidt, Christine V. Saunders, Chloë E. George, Thomas G. Scorer, Lynn M. R. McCallum, Tracey E. Madgett

**Affiliations:** ^1^ Faculty of Health – Drake Circus University of Plymouth, School of Biomedical Sciences Plymouth UK; ^2^ Component Development and Research Laboratory Welsh Blood Service Talbot Green, Pontyclun UK; ^3^ University Hospitals Plymouth Plymouth UK; ^4^ Centre of Defence Pathology Royal Centre for Defence Medicine Birmingham UK

## Abstract

**Background:**

Cryopreservation extends the shelf life of red blood cells (RBC) from weeks to years, offering major advantages for blood banking logistics, especially in remote environments. However, current glycerol‐based methods, though effective, are limited in scope due to complex post‐thaw washing and incompatibility with routine clinical workflows. This study evaluated plasma‐derived albumin as a potential alternative cryoprotective agent (CPA) for human RBC.

**Study Design and Methods:**

Donor‐derived RBC were suspended in 25% w/v bovine serum albumin (BSA) prepared in various diluents and tested for biocompatibility and cryoprotective efficacy under rapid freezing (immersion in liquid nitrogen) and gradual freezing with storage at −80°C. Post‐thaw recovery was quantified by measuring hemolysis after resuscitation of frozen cells to 37°C.

**Results:**

Albumin‐based media were non‐toxic, maintaining >99% RBC recovery prior to freezing. When rapidly frozen, albumin conferred strong cryoprotection (≥95% recovery) in salt‐rich diluents such as SAG‐M, Hartmann's, and Alsever's solutions, exceeding that of glycerol‐based CPA (77 ± 25%). Albumin's cryopreservative efficacy was enhanced in ionic media of neutral pH but was absent entirely under gradual freezing (<20% recovery).

**Discussion:**

The cryoprotective effect of albumin appears dependent on rapid cooling and ionic co‐solutes, suggesting a mechanism rooted in colloid–ion interactions rather than direct inhibition of ice formation. The dependence on freeze rate and ionic environment suggests that multiple solution properties contribute to its efficacy. While further work is required to clarify underlying mechanisms, albumin‐based methods may provide a basis for a simplified method of cryopreservation.

**Abbreviations**
%: percent
ANOVA: analysis of variance
ATD: adult therapeutic dose
BSA: bovine serum albumin
CPA: cryoprotective agent
CPD: citrate‐phosphate‐dextrose anticoagulant
Glu: glucose
HBSS: Hank's Balanced Salt Solution
HCT: haematocrit
HSA: human serum albumin
LN2: liquid nitrogen
Nacl: sodium chloride
PBS: phosphate buffered saline
RBC: red blood cells
SAG‐M: Saline‐Adenine‐Glucose‐Mannitol solution
Soln: solution
T: temperature
t: time
UK: United Kingdom

## 1 INTRODUCTION

While hypothermic storage (2–6°C) and improvements in the formulation of storage media have greatly extended the shelf‐life of cellular components, donor‐derived red cell units (RBC) are still only viable for 35 days from donation in the United Kingdom (UK), which presents a substantial challenge to the wider logistics of transfusion‐based medicine.^1^, ^2^ Cryopreservation is an alternative to the traditional method of refrigerated storage, where materials are preserved at ultra‐low temperatures, typically maintained by freezers or condensed gases (liquid nitrogen; LN_2_).^3^ As a method, cryopreservation prolongs the “shelf life” of cellular components on a scale of years to decades.^4^ However, effective cryopreservation requires cellular structures to be protected from the formation of crystalline ice, with biologically available water retained during the freezing process to avoid cellular lysis and incursion of ice‐mediated injury (cryoinjury).^5^, ^6^ Thus, cryopreservation requires the use of cryopreservatives (CPA) to maintain cellular integrity during freezing and subsequent thawing.^7^ These CPA are applied to cells prior to freezing and work by maintaining a biologically tolerable state within the cell and at membrane/fluid interfaces.^4^, ^6^, ^7^

Glycerol is the most widely utilized CPA, used globally in the cryopreservation of various cell types, including RBC.^4^ As a CPA, glycerol works by interrupting the normal hydrogen‐bond network of solvent water, introducing a thermodynamic and physicochemical barrier to freezing.^8^, ^9^, ^10^ In this role, glycerol is highly effective in preserving both the physical and functional features of RBC, with glycerol‐based methods of cryopreservation currently used to maintain stocks of rare phenotype RBC units in the UK and abroad.^11^, ^12^, ^13^ However, cryopreservation has yet to be adopted for routine component storage and remains an under‐utilized technology within transfusion medicine.

Conceptually, frozen RBC offer unique advantages to maintaining access to transfusion‐based interventions in austere environments or remote clinical theaters.^14^, ^15^, ^16^, ^17^, ^18^ Significant extension of a blood component's shelf life allows for greater stock resilience and eliminates the need for routine resupply due to time‐expiry of product.^1^, ^18^ The advantages of frozen blood components have already been established in maintaining effective transfusion‐based care in the austere environment^14^, ^19^; with the potential for positive impact in the civilian sector by allowing a build‐up of compatible‐type stock during periods of reduced demand in anticipation of events where demand spikes and/or donor activity becomes reduced.^1^, ^11^, ^15^

In practice, frozen RBC components cannot fulfill these idealized applications due to the logistical and technological limitations imposed by the process of cryopreservation.^20^ Despite the widespread use of glycerol for rare RBC phenotypes, it is impractical for mainstream blood banking use. This is due to the time‐consuming post‐thaw washing that is required to remove the glycerol from the RBC before they can be transfused, as high levels of glycerol can be toxic.^20^ These challenges represent a major hurdle to expanding the scope of cryopreservation to routine clinical use.^21^ The ideal system would address these challenges and fit within current manufacturing and banking infrastructure, to give a component that is stable under practical storage and transport conditions and be easily preparable by hospital/blood bank staff.

Following previous work exploring novel cryopreservatives,^22^ we examined plasma‐derived albumin as a cryopreservative for specific application in long‐term storage of RBC. Albumin has not been recently examined as a cryopreservative itself but rather as a supplement to other cryopreservative agents.^23^, ^24^, ^25^, ^26^ The aim of this work was to explore the viability of an albumin‐based method of cryopreservation of RBC for specific use within the scope of transfusion medicine. To this effect, we report the biocompatibility of albumin‐based cryopreservative media with RBC and its effectiveness in preventing cryoinjury of donor‐derived RBC using bovine serum albumin (BSA) as a cost‐effective stand‐in for human serum albumin (HSA). While the use of BSA as a proxy for HSA limits the translatability of this study, similarities between these orthologues at the molecular level allow for reasonable confidence that results derived from this study can be reproduced at later stages with HSA.

In the interest of exploring application towards unit‐scale cryopreservation, we also describe the current limitations of albumin‐based methods and relate those to the current manufacturing and component handling practices.

## 2 MATERIALS AND METHODS

### 2.1 Red blood cells

Human donor‐derived RBC were manufactured by the Welsh Blood Service from volunteer non‐remunerated whole blood donors with consent for collections to be used in research. Ethical approval for the study was received from the University of Plymouth Faculty of Health Research Ethics and Integrity Committee (Reference: 4683).

Donor whole blood was collected using Macopharma LQT (PVC/DEHP, bottom‐and‐top) or FQE (PVC/DEHP, top‐and‐top) systems with 66.5 mL citrate–phosphate‐dextrose anticoagulant (CPD; Macopharma, Tourcoing, France). Donations were processed on Day 1 following overnight ambient hold. FQE units were leucodepleted prior to centrifugation; LQT red cells were leucodepleted post‐separation. Whole blood was centrifuged at 3405 × *g* for 15 min at 22°C (Sorvall BP12+, Thermo Scientific, USA), and components were separated on G5 Plus devices (Fresenius Kabi, Bad Homburg, Germany). Final RBC concentrates contained 105 mL Saline‐Adenine‐Glucose‐Mannitol solution (SAG‐M).

RBC were prepared for analysis by washing three times with 0.1 M phosphate buffered saline (PBS; pH 7.0, 0.1% w/v sodium azide, Sussex Biologicals; Hailsham, UK) following centrifugation at 2000 *g* for 10 min. All washing steps were isovolumetric and preserved the hematocrit (HCT) of the original donor product. HCT for washed RBC was determined by methods described elsewhere.^27^ All experiments were conducted on RBC within 7 days from donation.

### 2.2 Cryopreservatives

Albumin‐based cryopreservative media was comprised of 25 g bovine serum albumin (BSA; ethanol fractionated, Millipore‐Sigma, UK) per 100 mL of cryopreservative diluent (Table 1). Glycerol‐based cryopreservative media was obtained commercially (S.A.L.F.; S.p.A. Laboraorio Farmacologico, Bergmao, Italy) as a sterile solution and used without additional processing. The composition (per liter dH_2_O) was as follows: 57.1 g glycerol, 2.67 g sodium lactate, 30 mg potassium chloride, 86 mg monobasic sodium phosphate monohydrate, and 227 mg dibasic sodium phosphate dodecahydrate.

**TABLE 1:** Compositional detail of cryopreservative diluents used in this study.

Cryopreservative diluent	Composition per L *d*H_2_O
0.9% w/v NaCl	9 g sodium chloride
0.18% w/v NaCl, 4% w/v glucose	1.8 g sodium chloride 40 g anhydrous d‐glucose
20% w/v glucose	200 g anhydrous d‐glucose
Alsever's solution (modified)	8 g anhydrous trisodium citrate 4.2 g sodium chloride 20 g anhydrous d‐glucose 500 mg citric acid monohydrate
Hartmann's (Ringer's Lactate) solution	6 g sodium chloride 400 mg anhydrous potassium chloride 270 mg calcium chloride dihydrate 4.5 mL 70% sodium lactate (*aq*.)
0.15 M PBS, sodium‐base	9.048 g monobasic sodium phosphate dihydrate 13.06 g dibasic sodium phosphate anhydrous
0.15 M PBS, potassium‐base	7.895 g monobasic potassium phosphate anhydrous 20.995 g dibasic potassium phosphate trihydrate
Hank's balanced salt solution (HBSS) (modified)	185 mg calcium chloride dihydrate 98 mg magnesium sulphate anhydrous 400 mg potassium chloride 60 mg monobasic potassium phosphate anhydrous 8.0 g sodium chloride 48 mg dibasic sodium phosphate anhydrous 1 g anhydrous d‐glucose 350 mg sodium bicarbonate
SAG‐M	8.77 g sodium chloride 169 mg adenine 9.0 g anhydrous d‐glucose 5.25 g mannitol

### 2.3 Evaluation of cryopreservative biocompatibility

Intrinsic compatibility of diluents/cryopreservative media with RBC (biocompatibility) was evaluated as described in previous studies with modified volumes.^22^ Briefly, 500 μL of washed RBC were added to 1000 μL of CPA and incubated for an initial 30 min at 22°C followed by 24 h at 4°C ± 2°C. Cell‐free supernatant was isolated by centrifugation (2000 x *g* for 10 min) and used to estimate hemolysis after the 24 h at 4°C (see Section 2.5). Negative treatment controls were prepared for each biological replicate alongside treatment groups (Table 2).

**TABLE 2:** Detail of methods used to prepare experimental controls and analytical reference material used in this study.

Cryopreservative biocompatibility	
Negative treatment control	*Washed RBC (500 μL) were suspended in 1000 μL of PBS, incubated at RT for 30 min before storage at 4°C for 24‐h. Supernatant was then isolated and cell‐free hemoglobin measured as described in methods*.
Cryopreservative efficacy	
Negative (unfrozen) treatment control	*Washed RBC (500 μL) were suspended in 1000 μL of cryopreservative diluent and stored at 4°C for 24‐h following a 45‐min induction period at ambient (22°C) conditions. After 24‐h, samples were warmed in a water bath (37°C) for 10 min prior to being resuspended and supernatant isolated*.
Positive (frozen) treatment control	*Washed RBC (500 μL) were suspended in 1000 μL of cryopreservative diluent and inducted/frozen as described prior to storage at − 80°C for 24‐h. After which, samples were thawed in a water bath (37°C) for 10 min prior to being resuspended and supernatant isolated*.
Hemolysis assay references	
0% hemolysis standard	*Washed RBC (500 μL) were suspended in 1000 μL of PBS and incubated for 30 min at 22°C. Without any further incubation, the supernatant was isolated and cell‐free hemoglobin measured as described*.
100% (complete) hemolysis standard	*Washed RBC (500 μL) were suspended in 1000 μL of deionized water dH*_*2*_ *O and incubated for 30 min at 22°C. Complete hemolysis of the standard was ensured by pulse vortex mixing (6777 LSETM Vortex Mixer; Corning Ltd.; Flintshire, UK), at the highest setting for 5 min. The supernatant/hemolysate was isolated and cell‐free hemoglobin measured as described*.

### 2.4 Evaluation of cryopreservative efficacy

Stock CPA was prepared as 1000 μL aliquots in 2 mL Eppendorf‐style polypropylene vials suitable for immersion in LN_2_ (Product 1153,534, Fisher Scientific, UK). To each aliquot, 500 μL of washed RBC were added and allowed to incubate at 22°C for 45 min. Samples in “flash frozen” groups were then immersed in LN_2_ for 30–40 s before storing at −80°C. Samples in “gradually frozen” groups were placed in a mechanical freezer at −80°C. After 24 h, the CPA/RBC samples were removed and thawed in a water bath (37°C) for 10 min. After RBC were resuspended, the supernatant was isolated and used to estimate the rate of hemolysis. Positive and negative treatment controls were prepared for each biological replicate alongside treatment groups (Table 2).

### 2.5 Hemolysis assay

Hemolysis of RBC following CPA incubation/freezing/thawing was estimated as described previously.^22^ Briefly, 10 μL of isolated supernatant was diluted 1:20 with *d*H_*2*_O and absorbance measured at 540 nm against a *d*H_*2*_O blank.^27^

### 2.6 Freezing kinetics of unit‐scale and small‐volume samples

Core temperature changes over time were recorded with an H‐type thermocouple probe and data‐logger system (TinyTag; UK). The size of the probe module allowed for placement within a 600 mL capacity PVC/DEHP unit bag (Macopharma; Middlesex, UK), modified to allow for Luer access and probe connections while retaining a fluid seal. A vacutainer (BD, 10.05 mm internal diameter, 0.10 mm wall thickness) was modified in a similar fashion and allowed for placement of the thermocouple probe within a 2 mL volume of media such that the distance from vacutainer wall to probe was equal to the distance from vial wall to sample core of the small‐volume samples. The size of the thermocouple probe was the limiting factor in design of this probe and while larger than the 2 mL vials used in the RBC experiments, this setup offered reasonable compromise and allowed for representative measurement/observation of small‐volume freezing behavior to be made.

Three solutions were examined: *d*H_2_O, 40% v/v glycerol (*aq*.) and 25% w/v BSA in *d*H_2_O. Freezing behavior of each solution was observed under the following conditions: after 10 min at room temperature (between 20°C and 22°C), units were placed in a mechanical freezer set to −80°C and left for 16 h; individual units were laid flat in the freezer without any overwrapping or insulation. Freezing rate of samples immersed in LN_2_ was examined by immersing the sample‐volume probe in LN_2_ for 30 s before placement in sample racking in a −80°C freezer.

Temperature logger settings for gradual freeze experiments recorded observations at 30 s intervals and for flash frozen, at 1 s intervals.

### 2.7 Data analysis

Analysis of variance (ANOVA) was used to compare variances across the means of the control and treatment groups. Differences were considered significant between control and treatment groups where *p* values were less than 0.05. Statistical analysis and data derivatization was performed using GraphPad Prism (GraphPad Software, Boston, MA, Version 10.4.1(627)).

## 3 RESULTS

### 3.1 Unit hematocrit and density of cryopreserved cells

The initial HCT of donor RBC was 0.54 ± 0.09 (*n* = 4) and reduced to 0.16 ± 0.05 following addition of cryoprotective media/diluent. All freezing and biocompatibility studies were performed at this lower HCT.

### 3.2 Cryopreservative biocompatibility

**FIGURE 1:**
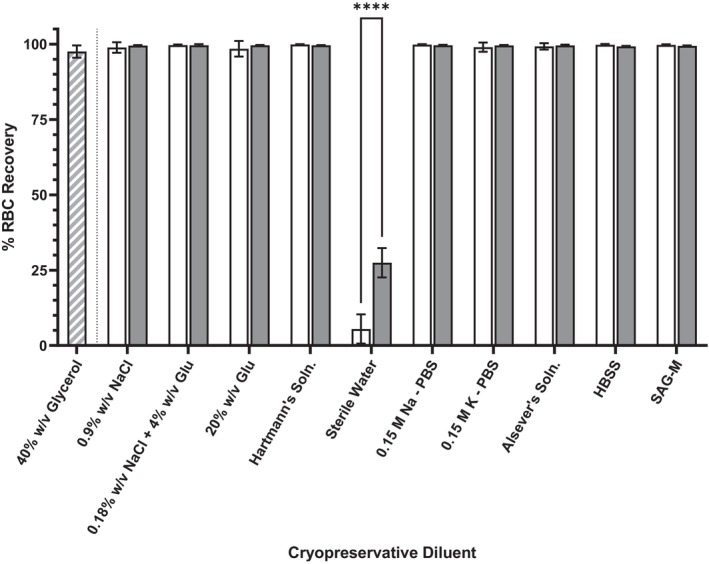
Biocompatibility of albumin and glycerol based cryoprotective media and cryopreservative diluent solutions (sodium chloride, NaCl; glucose, Glu; phosphate buffered saline, PBS; Hank's Balanced Salt Solution (Soln), HBSS; sodium adenine glucose mannitol solution, SAG‐M). Percent (%) red blood cell (RBC) recovery following 24 h incubation in cryopreservative diluent (negative cryopreservative control; white), diluent with 25 g per 100 mL bovine serum albumin (BSA; gray), or glycerol‐based cryopreservative media (gray‐white barred). Results shown are mean values derived from RBC from non‐repetitive donors (*n* = 4) with standard deviation indicated. Significance relative to control group as determined through analysis of variance (ANOVA) is indicated with respect to control values (****p < .0001).

For all diluent solutions examined, RBC recovery was ≥99.27% ± 1.04% between replicates (*n* = 4) except for sterile water where recovery was 5.49% ± 4.84% (Figure 1). With 25% w/v BSA, red cell recovery was ≥99.26% ± 0.15% apart from 25% w/v BSA in sterile water where recovery was 27.49% ± 4.86% (Figure 1). There was no significant change in recovery between any diluent and that diluent with 25% w/v BSA, with the exception of water (*p* = <.0005, Figure 1). Recovery of RBC in glycerol media was 97.56% ± 2.03% and was not significantly different from non‐water diluents and BSA‐supplemented media.

### 3.3 Efficacy in prevention of hemolysis due to rapid freezing

**FIGURE 2:**
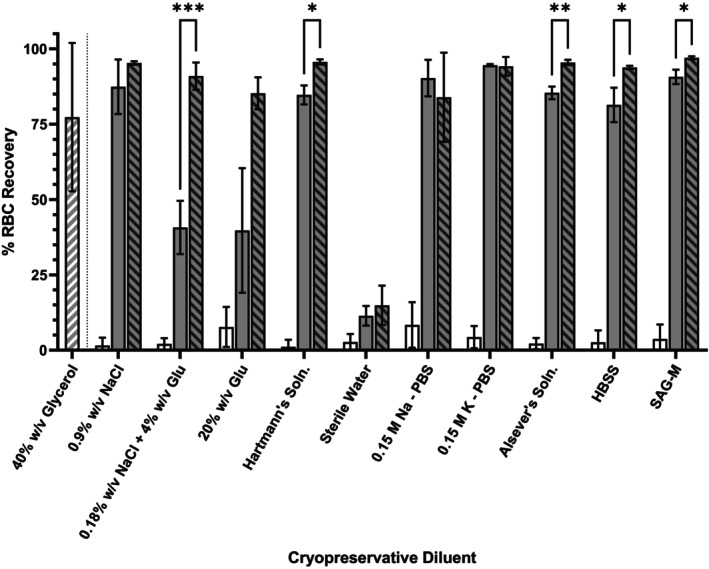
Cryopreservative efficacy of albumin‐ and glycerol‐based cryoprotective media and cryopreservative diluent solutions (sodium chloride, NaCl; glucose, Glu; phosphate buffered saline, PBS; Hank's Balanced Salt Solution (Soln), HBSS; sodium adenine glucose mannitol solution, SAG‐M) when flash‐frozen. Percent (%) red blood cell (RBC) recovery following immersion in liquid nitrogen (LN_2_) followed by 24 h at −80°C and rapid warming to 37°C. Samples suspended in cryopreservative diluent (negative cryopreservative control; white), diluent with 25 g per 100 mL bovine serum albumin (BSA) pH 5.0 (gray), diluent with 25 g per 100 mL BSA pH 7.0 (gray‐black barred) or glycerol‐based cryopreservative media (gray‐white barred). Results shown are mean values derived from RBC from non‐repetitive donors (*n* = 4) with standard deviation indicated. Significance relative to control group as determined through analysis of variance (ANOVA) is indicated with respect to control values (*p ≤ .05; **p ≤ .005; ***p ≤ .0005).

Where flash frozen, recovery of RBC in diluent solutions alone did not exceed 8.36% ± 7.56% across all replicates (*n* = 4), regardless of diluent (Figure 2). Recovery of RBC, where frozen in diluent supplemented with 25% w/v BSA, pH 5.0 was highest in potassium‐PBS (94.56% ± 0.35%, *n* = 4), sodium‐PBS (90.30% ± 6.07%, *n* = 4) and in SAG‐M (90.69% ± 2.38%, *n* = 4). Where frozen in 25% w/v BSA‐supplemented diluent, pH 7.0, RBC recovery was highest in SAG‐M (97.03% ± 0.52%, *n* = 4), Hartmann's solution (95.65% ± 0.86%, *n* = 4), and in Alsever's solution (95.48% ± 0.86%, *n* = 4). Modification of albumin‐based cryopreservative pH was found to have a significant impact on RBC recovery in 0.18% w/v NaCl + 4% w/v glucose (*p* = <.005), Hartmann's solution (*p* = <.05), Alsever's solution (*p* = <.005), HBSS (*p* = <.05), and SAG‐M (*p* = <.05) based media when comparing recovery from solutions where pH was 7.0 vs. pH 5.0. RBC recovery from glycerol‐based CPA media was 77.35% ± 24.61% (Figure 2).

### 3.4 Efficacy in prevention of hemolysis due to gradual freezing

**FIGURE 3:**
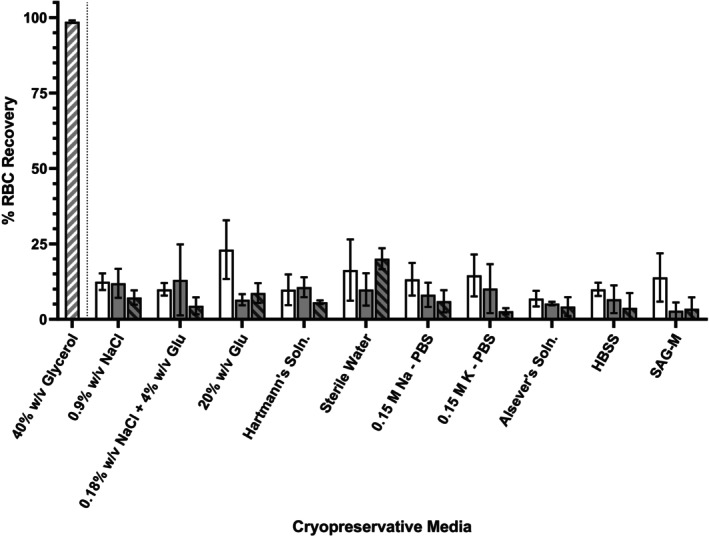
Cryopreservative efficacy of albumin‐ and glycerol‐based cryoprotective media and cryopreservative diluent solutions (sodium chloride, NaCl; glucose, Glu; phosphate buffered saline, PBS; Hank's Balanced Salt Solution (Soln), HBSS; sodium adenine glucose mannitol solution, SAG‐M) when gradually frozen. Percent (%) red blood cell (RBC) recovery following 24 h at −80°C and rapid warming to 37°C. Samples suspended in cryopreservative diluent (negative cryopreservative control; white), diluent with 25 g per 100 mL bovine serum albumin (BSA) pH 5.0 (gray), diluent with 25 g per 100 mL BSA pH 7.0 (gray‐black barred) or glycerol‐based cryopreservative media (gray‐white barred). Results shown are mean values derived from RBC from non‐repetitive donors (*n* = 4) with standard deviation indicated.

Where gradually frozen, RBC recovery in diluent solutions did not exceed 23.09% ± 9.74% between all replicates (*n* = 4) (Figure 3). Where frozen in the presence of 25% w/v BSA, RBC recovery did not exceed 13.06% ± 11.78% where CPA pH was 5.0 and did not exceed 20.05% ± 3.48% where CPA pH was 7.0. Pairwise comparisons between diluent and albumin‐supplemented groups found no instance where the presence of albumin had any significant impact on RBC recovery when gradually frozen. RBC recovery from glycerol‐based CPA media was significantly higher (98.61% ± 0.43%, *p* = <.0005) than all albumin‐based CPA media examined (Figure 3).

### 3.5 Kinetics of flash frozen, small‐volume samples

**FIGURE 4:**
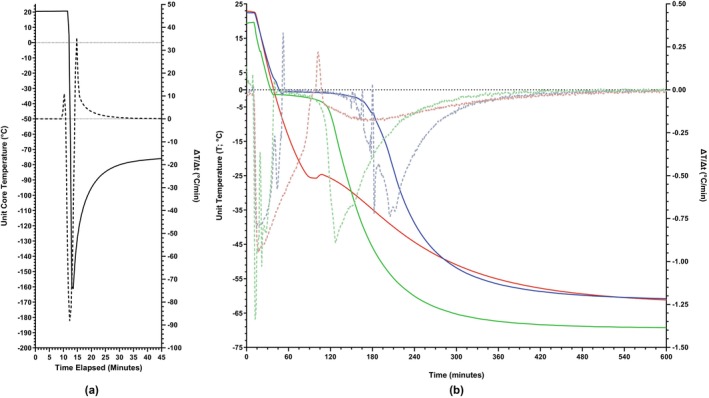
(A) Core temperature of a 2 mL volume sample (solid line, left axis) over a 45‐min period where, after 10 min at ambient temperature, the sample was immersed in liquid nitrogen (LN_2_) for 90 s before being placed in a mechanical −80°C freezer and allowed to equilibrate. Cooling rates and thermodynamic events are visualized by the first derivative of the temperature (*T*) vs. time (*t*) data (dashed line, right axis). Data obtained from technical duplicates. (B) Core temperatures of 350 mL units (solid lines, left axis) of albumin and glycerol solutions and *d*H_2_O over a 10‐h period where, after 10 min at ambient temperature, units were placed in a mechanical −80°C freezer and allowed to gradually freeze, unassisted and undisturbed. Solutions examined include sterile water (blue), 25% w/v bovine serum albumin (green), and 40% v/v glycerol (red). Cooling rates and thermodynamic events are visualized by the first derivative of the temperature (*T*) vs. time (*t*) data (dashed lines, right axis). Data obtained from technical duplicates.

When a representative small‐volume (2 mL) sample was frozen by immersion in LN_2_, the core temperature cooled at a maximal rate of ~ −90°C/min and reached −160°C within 90 s (Figure 4A). There was no observable difference in the freezing trace kinetic behavior between *d*H_2_O, 40% v/v glycerol, and 25% w/v BSA.

### 3.6 Kinetics of gradually frozen 350 mL units

When allowed to freeze passively by placement at −80°C, the solutions examined all undergo an initial period of supercooling, achieving a maximal cooling rate of −1°C/min (Figure 4B). The extent of supercooling is greater in 40% v/v glycerol and the solution continues to supercool until a core temperature of −27°C is achieved, at which point freezing occurs (as evidenced by the inflection point in the derivative trace, Figure 4B) and the rate of subsequent cooling is greatly reduced (to about −0.2°C/min).

The initial period of supercooling in both *d*H_2_O and 25% w/v BSA is interrupted when core temperatures drop a few degrees below 0°C where both solutions undergo freezing before continuing to supercool and equilibrate between −60°C and −80°C (Figure 4B). From the freezing trace and first derivative, the duration of the freezing transition in 25% w/v BSA is considerably shorter (1 h where Δ*T*/Δ*t* <0.1°C/min) than *d*H_2_O (1 h 30 min where Δ*T*/Δ*t* <0.1°C/min), with both solutions continuing to cool at a maximal rate of −0.7°C/min. All solutions reach a stable core temperature after 9 h (where Δ*T*/Δ*t* <0.1°C/min).

## 4 DISCUSSION

In the interest of developing a more practical method of RBC cryopreservation for routine clinical use, this study provides a preliminary examination of serum‐derived albumin as a CPA for specific use in human RBC. While albumin has been previously examined as a supplement to cryopreservative media,^24^ there has been no recent work which explores albumin as the primary CPA in cryoprotective media and historical studies lack sufficient detail to maintain relevance amidst contemporary methods and practices.^23^, ^24^ We examined the viability of albumin as an effective CPA when prepared in a range of RBC‐compatible solutions, including clinical infusion compatible media (0.9% w/v NaCl, 0.18% w/v NaCl with 4% w/v glucose, 20% w/v glucose, Hartmann's solution), solutions routinely used in RBC analysis in vitro (PBS, Alsever's solution, HBSS), and media used in the manufacture of RBC components (SAG‐M). All albumin‐based cryopreservative media examined (with the exception of 25% w/v BSA in *d*H_2_O) were found to be biocompatible with RBC with less than 1% hemolysis over 24‐h at 4°C. However, all albumin‐based media were examined for potential cryopreservative efficacy.

Albumin consistently failed in preventing hemolytic cryoinjury when albumin‐protected RBC were frozen gradually, with post‐thaw recovery not exceeding 20% across all solutions tested. In contrast, when rapidly frozen by immersion in LN_2_, albumin was highly effective at preventing cryoinjury. The dependence of CPA efficacy on freezing rate is a known phenomenon with optimal freezing rates varying between CPA.^28^, ^29^ Glycerol, as an example, has varied efficacy as a cryopreservative under varied freezing rates, with higher concentrations of glycerol being more effective at preventing RBC cryoinjury when frozen gradually.^28^, ^29^ The dependence by albumin on rapid freezing presents an obvious limitation for transitioning to larger volumes, such as an adult therapeutic dose (ATD) of RBC, where rapid freezing becomes increasingly difficult to the point of impracticality.^30^ This freeze‐rate dependence, however, strongly suggests that the protective mechanism of albumin is linked to conditions that are specific to end‐states produced by rapid freezing.

It is generally appreciated that solutions frozen rapidly do not “freeze” in the literal sense but instead undergo a glass‐transition (i.e., vitrify) where the solvent phase adopts thermodynamic and physical properties of a solid but retains the disordered molecular orientation of a liquid.^30^, ^31^, ^32^, ^33^ This is in stark contrast to media frozen gradually where prolonged freezing is allowed to progress, predominantly in the extracellular spaces. Thus, gradual freezing of cellular media results in an end‐state where large ice crystals are separated by hypertonic regions of unfrozen media.^28^, ^34^, ^35^ Additionally, gradual freezing of a cellular suspension achieves dehydration, as water is removed from solution by its sequestration into crystalline ice.^28^, ^35^ Our thermal traces of large‐ and small‐volume freezing support this interpretation: the plateau observed in *d*H_2_O and 25% w/v BSA during gradual cooling of a 350 mL unit reflects the latent heat of fusion and active crystallization of extracellular water, a process absent in flash‐frozen small volumes where conduction‐driven cooling supersedes crystallization. The lack of substantial RBC recovery when gradually frozen shows that the protective effect of albumin is incompatible with the phase changes and end‐state of an extensively frozen system and thus unable to prevent the two dominant injury pathways under these conditions: (i) freeze‐associated osmotic imbalance and cellular dehydration, and (ii) physical rupture of membranes caused by large extracellular ice crystals. Instead, its efficacy appears restricted to the context of vitrification, where these lethal mechanisms are suppressed.

In exploring different diluent media, we identified the presence of co‐solutes as a secondary determinant of the cryopreservative efficacy of albumin. Our study shows none of the diluent solutions examined were in themselves cryoprotective; it is only with the addition of 25 g per 100 mL BSA that substantial RBC recovery was achieved. Likewise, our inclusion of *d*H_2_O as a diluent allowed us to examine the cryopreservative efficacy of albumin in the absence of co‐solutes and found albumin itself is non‐cryoprotective. This effect suggests that the mechanism by which albumin prevents cryoinjury is dependent on the presence of co‐solutes and is influenced by their identity. Albumin prepared in salt‐rich solutions (normal saline, PBS, Hartmann's solution, Alsever's solution, SAG‐M) yielded the highest rates of recovery when vitrified while albumin in glucose‐rich media (0.18% w/v NaCl + 4% w/v glucose, 20% w/v glucose) showed markedly reduced efficacy, indicating that the role of the co‐solutes may be dependent on the presence of ionic species.

Our work also shows that the pH of the media further influences cryoprotective efficacy. Albumin rich media tested at a pH near albumin's isoelectric point (pI; pH 5.0)^36^ was consistently less effective than identical solutions at neutral pH (pH 7.0), with recovery significantly improved in several media (glucose‐rich solutions, Alsever's, HBSS, and SAG‐M) at pH 7.0. This observation implicates the charge state of albumin and suggests a role of electrostatic interactions with ions and/or the RBC membrane in the cryoprotective mechanism of albumin.^26^, ^37^, ^38^, ^39^

We thus hypothesize that the cryoprotective activity of albumin arises from the colloid behavior of the albumin protein and resultant electrostatic interactions with the RBC membrane as opposed to a direct inhibition of ice crystal formation. Albumin is known to exert osmotic pressure within compartments, with nearly half of its physiological oncotic effect attributable to Gibbs–Donnan interactions that alter ionic distributions across semipermeable barriers.^37^, ^39^, ^40^, ^41^, ^42^ Its behavior is highly sensitive to pH, as protonation state alters the distribution of positively and negatively charged side chains across the protein surface.^37^, ^39^, ^40^, ^41^, ^42^ Although the pI describes a state of net zero charge, albumin still presents heterogeneous patches of positive and negative residues that can associate with ionic co‐solutes.^43^, ^44^, ^45^ Prior studies have demonstrated pH‐dependent interactions between albumin and monovalent ions such as sodium, potassium, and chloride.^43^, ^44^, ^45^ Our findings align with these observations: at neutral pH, where albumin carries a net negative charge, its associative interactions with salts appear to enhance protection, whereas at pH 5.0 these interactions are diminished.

Together, the freeze‐rate and solution composition data suggest that the protective mechanism of albumin is only relevant under vitrification, where ionic and colloid effects may stabilize cells against injury. Under gradual freezing, the dominance of dehydration and mechanical injury by large extracellular ice crystals overwhelms this protective mechanism.

From a translational standpoint, SAG‐M, Hartmann's solution, and normal saline offer the distinct advantage of being clinically approved for infusion,^46^ which would allow direct compatibility with transfusion practice. However, the freeze‐rate dependence represents a major obstacle for clinical implementation at unit scale, where vitrification of large RBC volumes is technically challenging. While the present study is unable to provide any certain evidence as to the precise mechanism by which albumin prevents freezing injury, current evidence is clear that the mechanism by which albumin prevents cryoinjury in RBC is only effective where cells are vitrified and in the presence of ionic co‐solutes (i.e., salts). We propose the species and density of co‐solvent ions is relevant to the cryopreservative mechanism and we suggest it is based in inter‐solute interactions and collective effects upon the RBC. While an albumin‐based method of RBC cryopreservation is attractive and would carry distinct advantages for wider clinical use, current limitations identified by this study (namely the freezing rate) present obstacles to use with larger volumes of RBC. Future work should aim to characterize the role of ionic, colloidal, and electrostatic factors to albumin's cryoprotective effect, identifying those interactions that stabilize cells during vitrification and otherwise fail under crystallizing conditions. Additionally, future work should aim to characterize the albumin‐vitrified state by identifying (a) the minimum concentration of albumin required to achieve a cryoprotective system, (b) the glass‐transition temperature of albumin solutions, and (c) exploring the effects and/or consequences of transient warming events on RBC frozen in albumin. These will better inform the practicality of using albumin at a larger scale. By establishing a mechanistic understanding, research in this field will be better informed to optimize albumin‐based cryopreservation strategies for clinical use.

## FUNDING INFORMATION

The funding was provided by the Welsh Blood Service (WBS) as a PhD studentship between the WBS and University of Plymouth.

## CONFLICT OF INTEREST STATEMENT

The authors have disclosed no conflicts of interest.

## Data Availability

The data that support the findings of this study are available from the corresponding author upon reasonable request.
